# Development of Potential Therapeutic Agents from Black Elderberries (the Fruits of *Sambucus nigra* L.)

**DOI:** 10.3390/molecules29132971

**Published:** 2024-06-22

**Authors:** Yulin Ren, Gunnar Meyer, Andrew T. Anderson, Kaitlyn M. Lauber, Judith C. Gallucci, Gary Gao, Alan Douglas Kinghorn

**Affiliations:** 1 Division of Medicinal Chemistry and Pharmacognosy, College of Pharmacy, The Ohio State University, Columbus, OH 43210, USA; meyer.1336@buckeyemail.osu.edu (G.M.); anderson.4454@osu.edu (A.T.A.); lauber.51@buckeyemail.osu.edu (K.M.L.); gallucci.1@osu.edu (J.C.G.); 2 OSU South Centers, The Ohio State University, Piketon, OH 45661, USA; gao.2@osu.edu; 3 Department of Horticulture and Crop Science, College of Food, Agricultural, and Environmental Sciences, The Ohio State University, Columbus, OH 43210, USA

**Keywords:** elderberries, Sambucol^®^, phenolic constituents, antioxidants, antiviral, antitumor, therapeutic agents

## Abstract

Elderberry (*Sambucus nigra* L.) is a widespread deciduous shrub, of which the fruits (elderberries) are used in the food industry to produce different types of dietary supplement products. These berries have been found to show multiple bioactivities, including antidiabetic, anti-infective, antineoplastic, anti-obesity, and antioxidant activities. An elderberry extract product, Sambucol^®^, has also been used clinically for the treatment of viral respiratory infections. As the major components, phenolic compounds, such as simple phenolic acids, anthocyanins and other flavonoids, and tannins, show promising pharmacological effects that could account for the bioactivities observed for elderberries. Based on these components, salicylic acid and its acetate derivative, aspirin, have long been used for the treatment of different disorders. Dapagliflozin, an FDA-approved antidiabetic drug, has been developed based on the conclusions obtained from a structure–activity relationship study for a simple hydrolyzable tannin, β-pentagalloylglucoside (β-PGG). Thus, the present review focuses on the development of therapeutic agents from elderberries and their small-molecule secondary metabolites. It is hoped that this contribution will support future investigations on elderberries.

## 1. Introduction

*Sambucus* (Viburnaceae) species have historically been used as foods and medicinal herbs, of which *S. nigra* L. (black elder), *S. ebulus* L. (dwarf elder), and *S. sieboldiana* L. (Japanese red elder), are the most highly investigated. Black elderberry (*S. nigra* L.), also called elderberry, black elder, and European elder, is a deciduous shrub growing widely not only in Europe but also in Asia, Africa, and North and South America. This species has three subspecies, including the American elderberry (*S*. *nigra* ssp. *canadensis*), the European elderberry (*S*. *nigra* ssp. *nigra*), and the blue elderberry (*S*. *nigra* ssp. *cerulea*), of which the European elderberry is widely used commercially [1,2,3]. The present review focuses on the fruits of *Sambucus nigra* L. (elderberries) and their small-molecule secondary metabolite constituents, with the potential development of therapeutic agents from these natural products being discussed.

Elderberries are rich in vitamins and antioxidants that are beneficial to human health and hence, have long been used in different food products [4]. The most extensively utilized elderberries are produced from wild-growing elderberry shrubs, which contain more bioactive components than their cultivars [5]. However, the number of cultivars of elderberry is increasing currently. For example, elderberry has been successfully cultivated on more than 1600 acres in ten states in the U.S. (2022 USDA Census of Agricultural, https://www.nass.usda.gov/Publications/AgCensus/2022/index.php, accessed on 17 April 2024). Also, several other countries, including Hungary, Germany, and France in Europe, and China and Vietnam in Asia, are now major suppliers of this species. American elderberry and European elderberry are responsible for the majority of commercial elderberry production, but these two cultivars have different secondary metabolite profiles [6]. It has been well demonstrated that fully ripe elderberries contain more anthocyanins than partially ripe berries [7], thus the harvesting of these cultivars can be monitored by the accumulation of soluble solids in the berries and their color changes [8]. In addition, both the genotype and fertilization regime affect the phytochemical profiles and the antioxidant potential of the Greek *S. nigra* cultivar [9]. Climatic conditions have been found to have a great impact on the chemical components of cultivars of elderberries [10], while the content of the phenolic components of elderberry juice can vary during storage [11,12]. Thus, the genotype, fertilization regime, climatic conditions, and their storage can all affect the production of elderberries.

**Figure 1:**
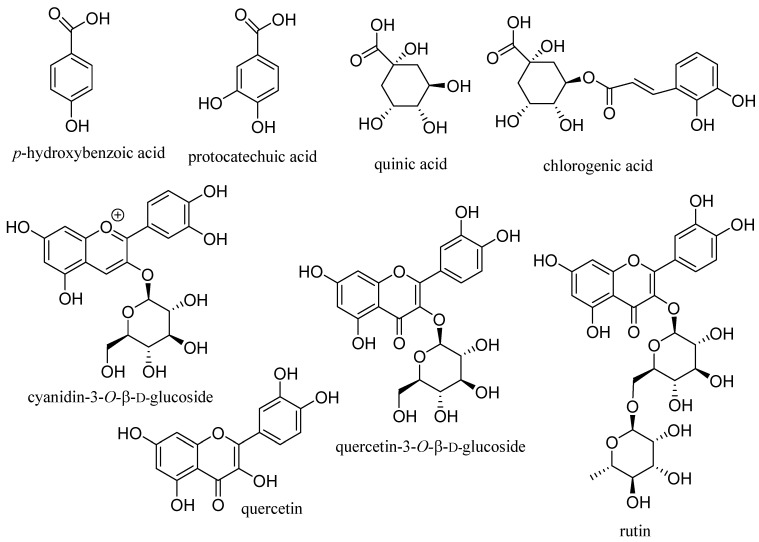
Structures of the major constituents identified from elderberries.

Elderberries contain relatively high levels of numerous phenolic compounds, including the major small phenolic acid components, *p*-hydroxybenzoic acid, protocatechuic acid, quinic acid, and chlorogenic acid; the anthocyanin, cyanidin-3-*O*-β-D-glucoside; other flavonoids, such as quercetin, quercetin-3-*O*-β-D-glucoside, and rutin, and tannins (Figure 1) [3,13,14,15]. The content of these major bioactive components is relatively high. For example, elderberry wine (per mL) contains 4.60 mg of phenolic compounds (gallic acid equivalent), 0.48 mg of anthocyanins (cyanidin-3-*O*-β-D-glucoside equivalent), 0.14 mg of other flavonoids (rutin equivalent), and 3.48 mg of tannins (catechin equivalent) [13].

The contents of the major phenolic compounds in elderberry wine (per mL) were reported to be as high as 42.96 μg of *p*-hydroxybenzoic acid, 52.46 μg of protocatechuic acid, 1674.77 μg of quinic acid, 17.67 μg of chlorogenic acid, 43.35 μg of quercetin, and 17.67 μg of quercetin-3-*O*-β-D-hexoside [13]. The large amounts of these components present would be expected to contribute to the biological activities observed for elderberries, as well as promote the potential development of therapeutic agents from these berries. Also, their potent antioxidative activity is supportive of other biological properties documented for elderberries, including antidiabetic, anti-infective, antimutagenic, immunomodulatory, and cardio-, gastro-, hepato-, and radioprotective effects [16,17].

Recently, several review articles on elderberries have been published, which have focused on the antiviral activity of elderberries [18], including effects on respiratory viral infections [19,20], specifically against influenza [21,22] and COVID-19 [23,24]; on the use of elderberries in the food industry [25]; on the chemical components and pharmacological activities of the berries [4,13,14], fruits, and flowers [26,27,28,29], as well as the whole plant of this species [2,3,15,16], and on the constituents and the female reproduction-focused bioactivity of the whole elderberry plant [30]. To complement these previous reviews, the present contribution highlights the development of potential therapeutic agents from elderberries and their small-molecule constituents. Discussed are also the mechanisms of action, clinical trial studies, and representative drugs used clinically. It should provide some important information to support future investigations on elderberries.

## 2. Elderberries Show Potent Antioxidant Activity and Have Been Used as Functional Foods

It has been well documented that elderberries show potent antioxidant activity through different mechanisms [14,15]. These include total antioxidant activity, as tested by a phosphomolybdenum method; radical-scavenging activity against 1,1-diphenyl-2-picrylhydrazyl radicals (DPPH) and 2,20-azinobis(3-ethylbenzothiazoline)-6-sulfonic acid radical cations (ABTS), reducing power activity toward cupric ion-reducing capacity (CUPRAC) and ferric reducing antioxidant power (FRAP), and metal-chelating activity, as evaluated by a ferrous ion method [13,17]. This general type of activity has also been observed in a clinical trial study with eight healthy and non-smoking volunteers, including four men and four women. Increased levels of total plasma phenolic components were observed in these volunteers. When tested with the Trolox equivalent antioxidant capacity (TEAC) and the total radical trapping antioxidant parameter (TRAP) assays, the plasma antioxidant capacity of these volunteers increased significantly after ingestion of 400 mL of elderberry juice carried out over one hour [31].

The major phenolic components of elderberries are postulated to counteract oxidative stress to help lower blood pressure, reduce glycemia, boost the immune system, decrease cancer risk, and increase the activity of antioxidant enzymes. Thus, these antioxidants may be important in supporting human health by alleviating cancer, infections, heart disease, diabetes, and other conditions [16]. In addition, elderberries have been cultivated as a commercial crop for their use in baked goods, jelly, juices, nutrient supplements, syrup, and wine (https://extension.missouri.edu/media/wysiwyg/Extensiondata/Pub/pdf/agguides/agroforestry/af1017.pdf, accessed on 18 April 2024). They have also been used as functional foods that show promising biological activities. For example, elderberry juice inhibited the activity of several enzymes, including acetylcholinesterase (AChE), butyrylcholinesterase (BChE), α-amylase, α-glucosidase, and tyrosinase [32]. A byproduct of the production of elderberry juice, the press cake, exhibited antioxidant and α-amylase inhibitory activities [33]. Also, elderberry wine showed antioxidant activity and inhibitory effects on AChE, BChE, α-amylase, α-glucosidase, and tyrosinase [13]. AChE and BChE are involved in the development of Alzheimer’s disease, and α-amylase and α-glucosidase modulate carbohydrate metabolism to contribute to the occurrence of diabetes, while tyrosinase plays a key role in the synthesis of melanin and some other pigments that could lead to melanoma. Thus, these elderberry products could be supportive of the treatment of Alzheimer’s disease, diabetes, and melanoma [13,33].

## 3. Development of Potential Therapeutic Agents from Elderberries

Elderberries possess various bioactivities of benefit to human health, including antioxidant, anti-infective, cytotoxic, antidiabetic, cardiovascular, and neuroprotective activities [15,16,26,27,28,29]. Thus, these berries show some potential for the development of therapeutic agents, especially those supportive of therapies of cancer and infections, as discussed in the following paragraphs.

Extracts of elderberries have exhibited activity towards different human cancer cells. Of these, anthocyanin-enriched extracts of elderberries were found to show cytotoxicity against human A2780 ovarian, MCF-7 breast, and HCT116 colon cancer cells [34]. These extracts also exhibited growth inhibitory activity against PC-3 human prostate cancer cells, which was potentiated by the use of gold nanoparticles (AuNPs) [35]. Similarly, silver nanoparticles (AgSNs) of an extract of elderberries diminished the viability of dysplastic oral keratinocytes (DOKs), while they stimulated the proliferation of human gingival fibroblasts (HGFs) [36]. In addition, a water extract of elderberries potentiated the activity of keratinocytes but slightly inhibited the viability of fibroblasts, indicating that elderberries may be helpful in the prevention of some skin conditions [37]. Thus, an extract of elderberries was found to decrease the proliferation of murine B16-F10 melanoma and human SH-SY5Y neuroblastoma cells [38]. An extract of elderberries was also found to stimulate the ovarian secretion of 17β-estradiol and progesterone, indicating that these berries may regulate steroidogenesis in ovarian cells [39]. Furthermore, a non-polar ethyl acetate extract of elderberries showed cytotoxicity against human LoVo colon and MCF-7 breast cancer cells [40].

Mechanistically, silver nanoparticles (AgSNs) capped with an elderberry extract targeted mainly the induction of necrosis, autophagy, and DNA lesions to inhibit the proliferation of dysplastic oral keratinocytes (DOKs). These biological activities involve many cellular proteins, including the proapoptotic proteins, tumor suppressor protein (p53) and BCL-2 associated X protein (BAX); the antiapoptotic proteins, pan Akt (protein kinase B) and phosphorylated pan Akt (pan p-Akt); the autophagy marker, microtubule-associated protein light chain 3 variant B (LC3B), and the DNA lesion marker, γ H2A histone family member X (γH2AX). Thus, in DOKs treated with AgSNs, the expression of pan Akt was found to be diminished and the expression levels of p53 and BAX were reduced, while the expression of ɣH2AX increased [36].

**Figure 2:**
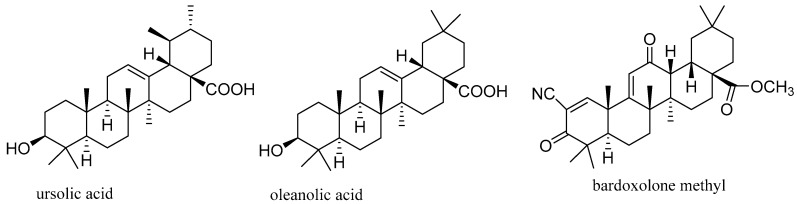
Structures of ursolic acid and oleanolic acid isolated from elderberries and a synthetic derivative of oleanolic acid, bardoxolone methyl (CDDO-Me), which has been evaluated in cancer clinical trial investigations.

Phytochemical studies showed that the triterpenoids oleanolic acid and ursolic acid (Figure 2) are the major cytotoxic components of the active extracts of elderberries. These triterpenes showed cytotoxicity against human LoVo colon and MCF-7 breast cancer cells, and the activity of ursolic acid was more potent than that of oleanolic acid, with its IC_50_ values being 7.7 and 10.7 μg/mL against LoVo and MCF-7 cells, respectively. However, these two triterpenes exhibited a similar activity toward normal human mammary epithelial hTERT-HME1 (ME16C) cells, indicating that their activity was not selective [40]. As discussed previously, the potential anticancer activity of ursolic acid has been evaluated in a phase I study to assess the multiple-dose tolerability, efficacy, and pharmacokinetics [41]. A new candidate anticancer agent, bardoxolone methyl (CDDO-Me or RTA 402 (Figure 2), has been developed from the synthetic modification of oleanolic acid. CDDO-Me has also been evaluated in several clinical trial investigations for its anticancer activity, from which a number of serious adverse effects were documented [41]. Thus, further modification of oleanolic acid, ursolic acid, and/or CDDO-Me is desired for the development of effective anticancer agents from these triterpenes. In addition, there are 28 clinical trial studies reported for CDDO-Me on https://www.clinicaltrials.gov/search?cond=CDDO-Me&page=3 website (accessed on 17 June 2024), which focused on cancer, cardiovascular diseases, diabetes, hepatic dysfunction, kidney diseases, and viral infection.

As discussed previously, elderberries have also shown potent inhibitory activity towards various types of infectious diseases [14,15,16,18,19,20,21,22,23,24,26], of which a large number of studies have focused on their antiviral activity, especially for their effects on respiratory viral infections, including influenza and COVID-19 [18,19,20,21,22,23,24]. The flavonoids, phenolic acids, and the immunomodulatory polysaccharides identified from these berries may contribute to their antiviral activity, but the mechanisms for this type of activity have not been investigated in detail. It has been proposed that elderberries could prevent influenza infection by competitively inhibiting the binding of the influenza virus to the host cells for their pathogenesis. Consistently, elderberries may mediate anti-COVID-19 activity by inhibiting the binding receptor–binding domain to the S protein ACE2-SARS-CoV-2. Also, these compounds may stimulate the host immune system to show antiviral activity [18,21]. For example, elderberries were found to show potent anti-influenza activity by affecting the post-infection phase and viral transmission and by modulating the release of the cytokines, interleukin-6 (IL-6), IL-8, and tumor necrosis factor (TNF) [42]. An aqueous extract of frozen fresh elderberries exhibited inhibitory activity against influenza A virus (A/H1N1), but it did not show any activities toward β-coronavirus-1 (HCoV-OC43) [43]. Furthermore, an anthocyanin-enriched extract of elderberries, Eldosamb^®^, reduced the secretion of the lipopolysaccharide (LPS)-induced TNF-α, and decreased interferon-γ (IFN-γ) release and increased IL-4 secretion when co-stimulated with the lymphocyte-specific stimulant and T-cell mitogen concanavalin A (Con A)/staphylococcal enterotoxin B (SEB). It thus stimulated the Th2-Helper cell response and mediated antiviral activity against the modified vaccinia Ankara (MVA) virus. These results indicate that Eldosamb^®^ may act as an immunomodulator by activating immune cells in a pro-inflammatory environment to mediate its antiviral activity [44].

The clinical trial investigations for the antiviral activity of elderberries have been summarized in a previous review [19]. Also, there are six clinical trial studies for elderberries posted at the website, https://www.clinicaltrials.gov/search?cond=ELDERBERRY, accessed on 25 April 2024 (Table 1). Elderberries exhibit promising anti-infective and immunostimulatory properties, based on which a trademarked extract product named Sambucol^®^ has been developed for the treatment of colds and influenza [Sambucol Black Elderberry Cold & Flu Relief Tablets, 30 CT (cvs.com), accessed on 18 April 2024]. Recently, consumption of Sambucol^®^ has increased greatly, owing to the COVID-19 pandemic, which sparked a wide interest in the enhancement of immunity with natural dietary supplements [45]. Sambucol^®^ has shown potent inhibitory activity toward the influenza virus. In Madin–Darby canine kidney cells, Sambucol^®^ inhibited the replication of human influenza virus type A, A/Beijing 32/92 (H3N2), A/Shangdong 9/93 (H3N2), A/Singapore 6/86 (H1N1) and A/Texas 36/91 (H1N1), human influenza virus type B, B/Ann Arbor 1/86, B/Panama 45/90, and B/Yamagata 16/88, as well as the animal influenza virus strains obtained from Northern European swine and turkeys, including A/Sw/Ger 2/81, A/Sw/Ger 8533/91, and A/Tur/Ger 3/91. It also reduced hemagglutination of the A/Beijing 32/92, A/Singapore 6/86, B/Panama 45/90, and B/Yamagata 16/88 virus strains [46]. Interestingly, Sambucol^®^ increased production of the inflammatory cytokines IL-1β, IL-6, IL-8, and TNF-α, and this activity was more potent than that observed for lipopolysaccharide (LPS) [47]. Following these, an in vivo study demonstrated that leishmania development was delayed when six- to eight-week-old female BALB/c mice were inoculated with *Leishmania major* and treated (gavage, two days later) with Sambucol^®^ (25 μL) six times, on alternate days, 2, 4, 6, 8, 10, and 12 post-infection. Such an effect could result from the immunomodulatory activity of Sambucol^®^ [48].

In a placebo-controlled double-blind clinical trial study for Sambucol^®^, during an outbreak of influenza B/Panama in 1993, a reduction of symptoms was observed. A complete cure from influenza infection was recorded by patients who consumed Sambucol^®^ daily for three days (four tablespoons for adults and two tablespoons for children) [46]. In addition, two clinical trial studies for the treatment of influenza and COVID-19 with Sambucol^®^, respectively, have been conducted (Table 1).

Sambucol^®^ activates the human immune system by inducing the production of inflammatory cytokines and shows potential immunoprotective and/or immunostimulatory activities in cancer and other ailments. Hence, this product could be used as a complementary tool in conjunction with other therapies for the prevention and/or treatment of infectious diseases and cancer. The further development of Sambucol^®^ as a possible therapeutic agent for the treatment of a wide range of ailments has been supported by the microencapsulation of extracts of elderberries, which improves the biochemical functionalities and therapeutic efficacy of these extracts [49].

## 4. Development of Potential Therapeutic Agents from the Major Second Metabolites of Elderberries

Elderberries contain both simple phenolic acids and more complex phenolic compounds, which account for their medicinal use. Of these, the most abundant compounds include *p*-hydroxybenzoic acid, quinic acid, chlorogenic acid, cyanidin-3-*O*-β-D-glucoside, quercetin, quercetin-3-*O*-β-D-glucoside, rutin, and tannins. Discussed in the following paragraphs is the potential development of these natural products as medicinal agents.

**Figure 3:**
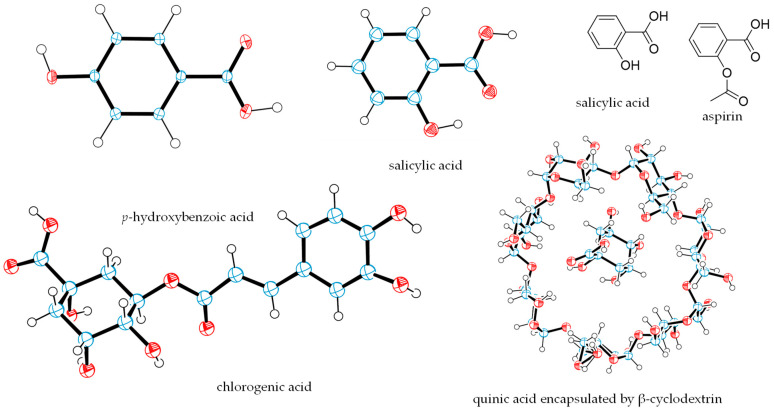
Crystal structures of *p*-hydroxybenzoic acid, salicylic acid, chlorogenic acid, and quinic acid encapsulated by β-cyclodextrin (β-CD) and the structures of salicylic acid and aspirin. The crystal structure plots were drawn using ORTEP-3 for Windows version 2020.1 [53], based on data from the literature. The ORTEP diagram for quinic acid shows one of the two complexes of quinic acid and β-CD, for which water molelcules are omitted, and the disordered region of the β-CD molecule is presented. In the ORTEP plots, oxygen atoms are red, carbon atoms are blue, and the small white circles represent hydrogen atoms, which are drawn with an artificial radius.

Benzoic acid is present widely in plant and animal tissues, and many of its derivatives have been used as antibacterial and antifungal preservatives in the food and pharmaceutical industries [50,51]. Of these, *p*-hydroxybenzoic acid is a major component of elderberry wine, and its content is as high as 42.96 μg/mL [13]. The structure of *p*-hydroxybenzoic acid was confirmed by its single-crystal X-ray diffraction data [52], for which a crystal plot was drawn using ORTEP-3 for Windows version 2020.1 (Figure 3) [53].

As summarized in a recent comprehensive review, *p*-hydroxybenzoic acid and some of its analogues showed multiple biological properties, including antialgal, antiestrogenic, anti-inflammatory, antimicrobial, antimutagenic, antioxidant, antiplatelet aggregating, antiviral, and hypoglycemic activities [54]. It was also found to show estrogenic activity and thus stimulated the growth of the estrogen-dependent MCF-7 and ZR-75-1 human breast cancer cells at a low concentration (10^−5^ M). However, this activity was less potent than that observed for 17β-estradiol (10^−8^ M), and, at a higher concentration (10^−3^ M), *p*-hydroxybenzoic acid reduced the proliferation of MCF-7 and ZR-75-1 cells [55].

Also identified in elderberries is salicylic acid [56], of which the crystal structure was determined by analysis of its single-crystal X-ray diffraction data (Figure 3) [57]. Salicylic acid has long been used as an antipyretic, anti-inflammatory, and analgesic agent, and it also modulates the human immune system in response to microbial infections [58]. Aspirin, an acetate derivative of salicylic acid, one of the most widely used drugs, is a non-steroidal anti-inflammatory agent for the treatment of fever, inflammation, and pain (https://en.wikipedia.org/wiki/Aspirin, accessed on 30 April 2024). Aspirin primarily targets the cyclooxygenase (COX) enzymes to mediate its bioactivities. These enzymes are frequently overexpressed in cancer cells, of which the inhibition affects angiogenesis and hence may inhibit cancer metastasis. Thus, aspirin shows some promising anticancer activity, as evidenced by its reduction of the incidence of various gastrointestinal malignancies and by its survival promotion, which has been observed in clinical studies. For example, regular use of aspirin reduced the incidence of hepatocellular carcinoma in patients with chronic liver disease [59,60].

Patients with diabetes have a high risk of cardiovascular disease, so the use of the antiplatelet agent aspirin is frequently recommended to mitigate such risk. However, a recent clinical trial study showed that the role of aspirin in the primary prevention of cardiovascular disease in diabetes patients was not established unambiguously, and further investigations were recommended [61]. A similar conclusion was obtained from another clinical trial study for older adults, in which aspirin was not found to affect incident cognitive or functional decline [62]. Thus, the use of aspirin for the prevention of cardiovascular diseases seems to be questionable. However, interest in the therapeutic effects of salicylic acid and aspirin on various diseases is increasing, as indicated by a large number of clinical trials being conducted (232 for salicylic acid and 1001 for aspirin (https://www.clinicaltrials.gov/website, accessed on 30 April 2024).

Quinic acid and chlorogenic acid are also major components of elderberries, with their content in elderberry wine having been reported as 1674.77 μg/mL and 17.67 μg/mL, respectively [13]. The structures of these compounds were confirmed by single-crystal X-ray diffraction data collected from crystals of the complexes of these compounds as well as from β-cyclodextrin, and their absolute configurations were determined based on those reported for β-cyclodextrin [63,64] (Figure 3).

Quinic acid shows various pharmacological properties, including antitumor, antidiabetic, antimicrobial, antinociceptive, and antioxidant activities, as discussed recently in a comprehensive review [65]. Chlorogenic acid exhibits similar activities and also regulates carbohydrate and lipid metabolism, and protects the liver, kidneys, and the nervous system [66]. As summarized previously, chlorogenic acid showed cytotoxicity against a panel of human cancer cells [29]. It inhibited the proliferation, migration, and invasion of cancer cells by targeting p53 and related proteins, p38 mitogen-activated protein kinase (p38 MAPK), c-Jun amino-terminal kinase (JNK), c-Myc, reactive oxygen species (ROS), and other proteins [66]. Chlorogenic acid functions as a safe differentiation inducer to inhibit solid tumor growth [67], and it also targets DNA methyltransferase 1 (DNMT1) to mediate such activity [68]. Interestingly, chlorogenic acid synergizes with doxorubicin for its activity against human U2OS and MG-63 osteosarcoma cells [69]. As discussed previously, several clinical trial investigations have been conducted for the therapeutic potential of chlorogenic acid against cancer and cardiovascular diseases [70], indicating that this phenolic substance shows some therapeutic potential for the treatment of these diseases.

Elderberry plants have one of the highest contents of anthocyanins, which have been used as natural coloring agents in the food industry, owing to their nutritional value and other biological properties [71]. Thus far, the structure of the aglycone of these anthocyanins, cyanidin, has been confirmed using the single-crystal X-ray diffraction data collected from cyanidin bromide monohydrate [72]. Anthocyanins show various biological properties, including antitumor, antidiabetic, anti-infective, anti-obesity, neuroprotective, and cardiovascular disease prevention activities [73]. These activities are closely similar to those observed for elderberries, indicating that these major components are the active constituents present [28,29]. Anthocyanins mediate their bioactivities by targeting mitogen-activated protein kinase (MAPK), nuclear factor κB (NF-κB), AMP-activated protein kinase (AMPK), and Wnt/β-catenin signaling pathways, as well as other important cellular processes [73]. In addition, these compounds were recently found to target the tumor lipid membrane to exhibit their antioxidant and antitumor activities [74].

In microorganism cells, structural changes to the lipid membranes affect the resultant biological functions. To mediate their biological activities, a compound needs to interact with the cell lipid membranes in order to transport into cells. Such interactions could result in changes in the lipid membranes, which then trigger and mediate the bioactivities of this compound. Furthermore, to transport into cells, this compound also needs to bind to human serum albumin (HSA), the main protein of blood plasma that plays many important roles in the human body, and such an interaction could affect the bioavailability of this compound. To understand the antioxidant and cytotoxic activities, the effects of an extract and the major anthocyanin component of elderberries, cyanidin 3-*O*-β-D-glucoside, on the lipid phase of tumor mimic membranes have been investigated. The results showed that both the extract and this anthocyanin increased the packing order in the hydrophilic region of the tumor mimic membranes, indicating that they may rigidify the membranes to protect them from oxidative stress. These natural products were also found to interact with HSA and quench its intrinsic fluorescence and hence, they inhibited the activity of COX-1 and COX-2 to mediate their anti-inflammatory properties and cytotoxicity against MCF-7 human breast cancer cells. Importantly, these products can be encapsulated with soy lecithin liposomes, which improve their stability. As a result, such liposomal capsules enhance the biological activities and bioavailability of the elderberry extract and cyanidin 3-*O*-β-D-glucoside. Thus, the lipid membranes could be the primary target of these elderberry products [74].

Currently, interest in anthocyanins is increasing, as indicated by a large number (102) of clinical trial investigations of their potential in the treatment of cancer, cardiovascular diseases, diabetes, and various infections (https://www.clinicaltrials.gov/search?cond=anthocyanidin&page=10, accessed on 2 May 2024).

In addition, elderberries produce further flavonoids, of which the content of quercetin in elderberry wine is as high as 43.35 μg/mL [13]. The structure of quercetin has been confirmed via powder X-ray diffraction and solid-state NMR spectroscopy [75]. Quercetin is a naturally occurring flavonoid used widely in the nutraceutical and food industries [76], and its antitumor and anti-infective properties have been reviewed extensively [29,77,78]. Quercetin was also found to show some biological properties in the contexts of wound healing and aging [79], as well as cardiovascular and neuroprotective properties [80,81,82], and potential therapeutic effects on epilepsy [83]. As summarized previously, several clinical trial investigations have been conducted on the therapeutic potential of quercetin, including its potential therapeutic effects against cancer, cardiovascular disease, diabetes, and infections [70]. Thus, quercetin shows some promise for the treatment of these diseases.

**Figure 4:**
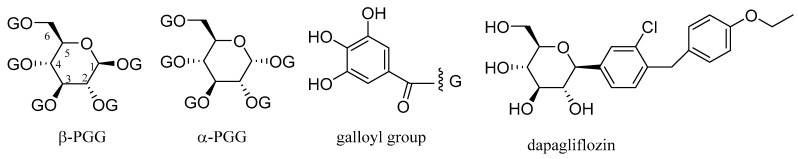
Structures of the antidiabetic lead compounds, α-PGG and β-PGG, and of dapagliflozin, an FDA-approved antidiabetic drug.

Tannins are also a major component of elderberries, and their content in elderberry wine has been reported to be 3.48 mg/mL [13,84]. These types of natural products include hydrolysable tannins (ellagitannins and gallotannins) and condensed tannins (proanthocyanidins and oligo-polymeric complex tannins). They are water soluble and show potential antitumor, antidiabetic, anti-infective, antioxidant, and cardiovascular and neuroprotective activities, for which the main targets have been postulated as the modulation of key enzymes and the activation of metabolic pathways and changes in the metabolic fluxes [85,86]. For example, a simple hydrolysable tannin, β-pentagalloylglucoside (β-PGG), was found to exhibit potential antidiabetic activity, and its isomer, α-PGG, was more active [87,88] (Figure 4).

As an insulin mimetic, α-PGG stimulated glucose transport in 3T3-L1 adipocytes by targeting the insulin receptor (IR) and the PI3K/Akt/GLUT4 pathway. It induces the phosphorylation of IRs and Akt, activates PI3K, and stimulates membrane translocation of GLUT4 to transport glucose [87]. A structure–activity relationship (SAR) study for α-PGG and its glucose transport stimulatory activity showed that both the glucose core and the galloyl group are necessary for α-PGG to mediate its activity, while glucose and gallic acid themselves are inactive. The galloyl group linked to the C-1, -2, -3, and -4 positions is essential, but that connected at the C-6 position is not important, indicating that α-PGG can be modified at the C-6 position. This has been supported by a prepared synthetic derivative, 6-chloro-6-deoxy-1,2,3,4-tetra-*O*-galloyl-α-D-glucopyranose (6Cl-TGQ), which showed more potent activity than α-PGG. Thus, the introduction of chlorine in the α-PGG molecule may improve its antidiabetic potential [88]. Furthermore, in vivo studies demonstrated that a sharp decline in blood glucose was found when healthy CD-1 mice (25 g) were injected with streptozotocin (STZ) (250 mg/kg) [for induction of type 1 diabetes mellitus (T1DM)] and then treated with 6Cl-TGQ (10 mg/kg orally, single dose). Also, a long-term reduction of blood glucose was observed when healthy C57BL/6J male mice were fed with high-fat diet (HFD) [for induction of type 2 diabetes mellitus (T2DM)] and then treated with 6Cl-TGQ (5 mg/kg orally, once every other day) for ten weeks. These results indicate that 6Cl-TGQ decreases the blood glucose levels in both STZ-induced T1DM and HFD-induced T2DM mouse models. Thus, 6Cl-TGQ could be a promising lead compound and shows some promise for the prevention and treatment of diabetes mellitus, including T1DM and T2DM. Mechanistically, 6Cl-TGQ selectively targets the insulin receptor signaling pathway to mediate its potential antidiabetic activity [89].

Consistent with these conclusions on the SAR, an antidiabetic agent, dapagliflozin [brand name, Farxiga (U.S.) or Forxiga (Europe), oral tablets] (Figure 4), has been developed successfully by Bristol Myers Squibb Company. Dapagliflozin was prepared from a glucose template, with a 4-chloro-3-[(4-ethoxyphenyl)methyl]phenyl group being introduced directly at the C-1 position. The glucoside formation through creation of a carbon–carbon bond protects dapagliflozin from degradation by glucosidases [90]. Differing from α-PGG and 6Cl-TGQ, which target the insulin receptor and function as glucose transport activators to mediate their antidiabetic potential, dapagliflozin reversibly inhibits sodium–glucose co-transporter 2 (SGLT-2) in the renal proximal convoluted tubule and obstructs the ability of the kidneys to reabsorb glucose to increase urinary glucose excretion and lower blood glucose levels. As a potent SGLT-2 inhibitor, dapagliflozin also reduces body weight, lowers blood pressure, and decreases the occurrence of cardiovascular incidents and renal complications in T2DM patients. As a result, this agent has successfully been used to treat diabetes, heart failure, and kidney disease [90,91,92]. Thus, dapagliflozin was approved by the FDA in 2014 for the treatment of diabetes (https://www.accessdata.fda.gov/drugsatfda_docs/nda/2014/202293Orig1s000TOC.cfm, accessed on 6 May 2024). It was also approved by the FDA for the treatment of heart failure in 2020 (FDA approves new treatment for a type of heart failure | FDA) and for the treatment of chronic kidney disease in 2021 (https://www.fda.gov/news-events/press-announcements/fda-approves-treatment-chronic-kidney-disease, accessed on 6 May 2024).

Previously, two pentacyclic triterpenes, oleanolic acid and ursolic acid (Figure 2), have been identified as the major cytotoxic components of elderberries, the contents of which in seedless dried elderberries are probably in the range of 0.05–0.15% [40]. The potential antitumor activity of these triterpenes has been discussed previously, and a new synthetic derivative of oleanolic acid, bardoxolone methyl (CDDO-Me), has been evaluated in clinical trial investigations for its potential anticancer activity [41]. More recently, a mixture of the major components of birch triterpenes used as a topical gel, oleogel-S10, has been evaluated in a double-blind, randomized, vehicle-controlled phase III study for its efficacy and safety in the treatment of epidermolysis bullosa. Oleogel-S10 was found to be well tolerated and it accelerated wound healing in this condition [93]. Thus, it was approved by the FDA in 2023 for the treatment of wound-associated epidermolysis bullosa [FDA Approves Birch Triterpenes Topical Gel for Treatment for Adult, Pediatric Patients With JEB, DEB (pharmacytimes.com), accessed on 6 May 2024). The major components of oleogel-S10 were identified as oleanolic acid, erythrodiol, lupeol, betulin, and betulinic acid [94]. Betulinic acid inhibited mitochondrial transmembrane potential, and oleanolic acid showed NF-κB inhibitory activity [95]. Epidermolysis bullosa is an inherited multisystem disease that is very difficult to treat, and the effectiveness of the therapeutic effects of oleogel-S10 on this disease indicates the considerable medicinal promise of these triterpenes.

## 5. Concluding Remarks

**Figure 5:**
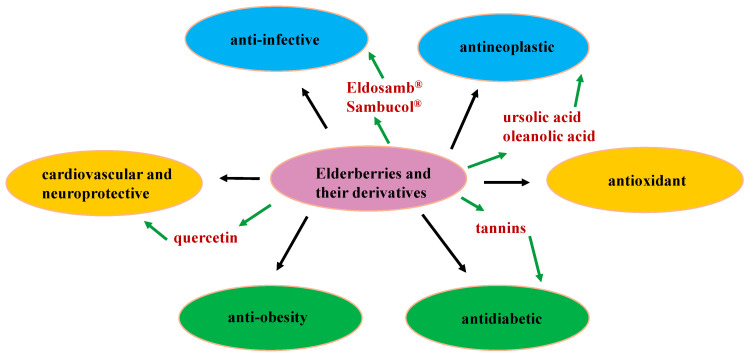
Examples of biological properties reported for elderberries and their major bioactive secondary metabolites.

Elderberries have long been used nutritionally and medicinally for the prevention and treatment of various diseases. Two extract products, Eldosamb^®^ and Sambucol^®^, have been developed as immunomodulators for the treatment of respiratory viral infections, including influenza and COVID-19 [44,46,47] (Figure 5). Eldosamb^®^ is a water-soluble anthocyanin-enriched extract that has been used as a colored reagent in the food and pharmaceutical industries, as it can also function as an optimal lead in improving the solubility of other pharmaceutical agents. Thus, the products derived from elderberries seem to possess some potential for further investigations on the development of new therapeutic agents when certain novel techniques are employed, including microencapsulation, which can improve their functionalities and therapeutic efficacy [49]. Also, elderberries are rich in bioactive phenolic compounds, including simple phenolic acids, anthocyanins and other flavonoids, and tannins. The high levels of these components contribute to the biological activities observed for elderberries themselves, in which several bioactive agents are present, including salicylic acid, chlorogenic acid, and quercetin. These components have been either used clinically for the treatment various ailments or evaluated in clinical trials for the potential development of new pharmaceutical agents, and they provide critical information supporting further investigations on elderberries. Thus far, phenolic compounds have been characterized as the major bioactive components of elderberries, but the identification of other types of compounds in these berries is comparatively limited. Thus, the identification of additional bioactive components in elderberries may support the further development of new therapeutic agents derived from these berries.

Synthetic modification can overcome major problems observed in the development of natural products as effective therapeutic agents, including limited solubility, poor bioavailability and efficacy, and undesired toxicity [96,97]. As discussed above, elderberries produce salicylic acid, from which aspirin, a well-established drug used widely for the treatment of various diseases, has been developed earlier. Oleanolic acid, one of the major triterpene components of elderberries, was modified synthetically, and CDDO-Me has been prepared. CDDO-Me showed potential antitumor activity and has been evaluated in several cancer clinical trial investigations [41]. As a potent activator of nuclear factor erythroid 2-related factor 2 (Nrf2), CDDO-Me can regulate neutrophil senescence during the progression of knee-joint damage [98], and it also reduced the progression of hemolytic anemia and endothelial function [99]. Nrf2 plays a role in kidney physiology and disease [100], thus CDDO-Me was evaluated in the CARDINAL trial, a phase 3 study in patients with Alport syndrome [101]. Following these, it could be possible to discover novel therapeutic agents from the synthetic modification of other major components of elderberries.

Natural products offer a valuable source for the discovery of new pharmaceutical agents, from which the new structures that have been characterized are specifically supportive of the design and discovery of new drug entities [102,103]. For example, tannins, one group of major components of elderberries, were found to show potential antidiabetic activity. In an earlier work, the antidiabetic lead compound, α-PGG, was identified. Subsequent structure–activity relationship investigations showed that both the glucose core and substitution at the C-1 position are critical for α-PGG to mediate its potential antidiabetic activity, which was enhanced by the introduction of a chlorine atom [87,88,89]. Based on these conclusions, an FDA-approved antidiabetic drug, dapagliflozin, has been developed using glucose as a template, with a chlorine-bearing phenyl moiety being substituted directly at the C-1 position [90,91,92]. This example indicates that other therapeutic agents may be developed from elderberry constituents.

The structures of natural products may provide some complex scaffolds, which could target specific proteins or signaling pathways to exhibit their bioactivities and thus support the target-directing drug design strategy [97]. In this regard, the inhibitory effects on AChE, BChE, α-amylase, α-glucosidase, and tyrosinase of elderberries could be employed in future drug design and discovery [13]. In addition, elderberry-derived water-soluble anthocyanins were found to target the MAPK, NF-κB, AMPK, and Wnt/β-catenin signaling pathways. Also, these phenolic compounds were recently demonstrated to interact with the tumor lipid membrane that plays a critical role in developing cancer [73,74]. Thus, elderberries could be used in the design of new cancer chemopreventive and chemotherapeutic agents by targeting the tumor membrane.

An inflammatory tumor microenvironment (TME) may be formed by a growing tumor surrounded by inflammatory immune cells, and communication between the stroma and malignant cells enables cancer cells to invade normal adjacent tissues. The TME is characterized by acidity, hypoxia, increased lactate and reduced glucose concentrations, secretome changes, and the recruitment of stromal and immune cells, and extracellular acidity could be formed by the deregulated energy metabolism of cancer cells to promote cancer progression [104]. Thus, interactions between any therapeutic agents and TME could contribute critically to the successful prevention and treatment of cancer [105]. Nrf2 acts as a master regulator of the response to oxidative stress and inflammation [106], in which oxidative stress plays a critical role in the development of cancer within the cellular microenvironment [107]. Thus, Nrf2 has a complex role in TME, and its activation can occur in tumor-associated macrophages (TAMs) and facilitate an anti-inflammatory, immunosuppressive tumor immune microenvironment (TIME) [108]. Thus, the potent Nrf2 activator, CDDO-Me, has been evaluated in several clinical trial investigations [41]. Elderberries show multiple biological activities, including antidiabetic, anti-infective, antioxidant, cancer-related, and immunomodulatory activities, which are all involved in the TME. This indicates that elderberry constituents could contribute to TME-targeted cancer control, of which further investigations could be supportive in the discovery of new therapeutic agents for the prevention and treatment of cancer. This is supported by the successful cultivation of elderberries, which should provide sufficient elderberry material required for the development of therapeutic agents.

## Figures and Tables

**Table 1:** Clinical trial investigations for elderberries and their major second metabolites reported on the website https://www.clinicaltrials.gov (accessed on 18 April 2024).

Year(s)	Clinical ID	Investigation Title
25 May 2023–26 November 2023	NCT05994586	The efficacy of an AP029 mix in patients with impaired carbohydrate metabolism
7 July 2021–2 September 2022	NCT05723497	Elderberries and obesity
1 September 2016–1 December 2019	NCT02414607	Effect of elderberry juice on cognition and inflammation in patients with mild cognitive impairment
29 January 2018–25 June 2019	NCT03410862	Evaluating the safety and clinical efficacy of elderberry extract in patients with influenza
10 October 2022–present	NCT05435144	Elderberry for immune support
1 September 2006–1 September 2009	NCT00375115	Efficacy of Sambucol^®^ in the treatment of influenza
5 January 2021–16 May 2022	NCT05489770	BERRY—a study of Sambucol^®^ in the treatment and reduction of symptoms in participants with COVID-19 (BERRY)

## Data Availability

Not applicable.

## Abbreviations

The following abbreviations are used in this manuscript:

AChE	Acetylcholinesterase
AMPK	AMP-activated protein kinase
BChE	Butyrylcholinesterase
β-CD	β-cyclodextrin
CDDO-Me	Bardoxolone methyl
6Cl-TGQ	6-Chloro-6-deoxy-1,2,3,4-tetra-*O*-galloyl-α-d-glucopyranose
COX	Cyclooxygenase
IFN-γ	Interferon-γ
IL	Interleukin
JNK	c-Jun amino-terminal kinase
LPS	Lipopolysaccharide
MAPK	Mitogen-activated protein kinase
MVA	Modified vaccinia Ankara virus
NF-κB	Nuclear factor κB
Nrf2	Nuclear factor erythroid 2-related factor 2
PGG	Pentagalloylglucoside
ROS	Reactive oxygen species
SAR	Structure–activity relationship
SGLT-2	Sodium–glucose co-transporter 2
T1DM	Type 1 diabetes mellitus
T2DM	Type 2 diabetes mellitus
TME	Tumor microenvironment
TNF	Tumor necrosis factor
